# Operation for Hernia

**Published:** 1860-07

**Authors:** F. B. Haller

**Affiliations:** Vandalia, Ill.


### ARTICLE 4.

### OPERATION FOR HERNIA.

BY F. B. HALLER, M. D., VANDALIA, ILL.

MESSRS. EDITORS: Having had an interesting case of hernia, to me, and thinking a brief account of it might interest you and the profession, I herewith send it to you to dispose of as you think best.

Was called, March 8th, 1860, twenty-five miles from our place, to see Daniel Boaz, aged 21, of a good constitution and temperate habits, who, the messenger said, had hernia that his attending physician could not reduce, and that he would have to be operated upon.

On my arrival I learned from the attending physician that he had diligently tried for two days to reduce the hernia by taxis, but with all his ingenuity had failed.

By this time four days had elapsed since it had become strangulated. Having obtained this history of the case, and diagnosed indirect inguinal hernia, I had him brought thoroughly under the aenesthetic influence of chloroform, when I made an effort by taxis to reduce, but finding it abortive, proceeded to cut down on the tumor, upon reaching the sack found it had become partially gangrenous, laying it open, discovered it was minus intestines it being merely a large portion of the omentum that had become strangulated, the part, at least one-half, that was gangrenous I cut off, dividing the stricture, put back all but a small portion that I retained in the wound, of the remainder. The wound was dressed in the usual manner. He bore the operation well. I left him six powders of calomel and opium, to be given at intervals of two or three hours, to be followed by oil. Since this he has been under the charge of his regular attending physician. He has recovered. The wound suppurated finely. I have written to his physician to send me a history of the case and its progress since the operation, and the treatment; but he has declined doing so, and I can only partly report. Suffice it to say say, he is one of that kind of practitioners who style themselves eclectics.

The points of interest in this case, to me, are, the length of time that had elapsed before operating, the violent taxis used, the amount of gangrened omentum cut off, and that he should recover.

